# Augmented reality for teaching undergraduate human anatomy: An educators' perspective

**DOI:** 10.1002/ase.70214

**Published:** 2026-03-08

**Authors:** Ally Williams, Zhonghua Sun, Mauro Vaccarezza

**Affiliations:** ^1^ Curtin Medical School Curtin University Perth Western Australia Australia; ^2^ Curtin Medical Research Institute (Curtin MRI) Curtin University Perth Western Australia Australia

**Keywords:** anatomy education, augmented reality, Australian university educators, health science, mixed reality

## Abstract

The purpose of this study was to explore the perspectives of Australian educators on using augmented reality (AR) as a method for learning human anatomy in the undergraduate health sciences. This will determine the current value of AR and guide future research and development. This prospective qualitative study used a mixed‐methods approach to gain detailed feedback from 10 anatomy educators at Curtin University. Educators interacted with mobile AR using an iPad and the Complete Anatomy application. A survey measured perceived usability through the System Usability Scale (SUS) and used Likert‐scale responses and short‐answer questions to determine educators' perspectives of AR. The SUS measured a mean usability score of 58.25, SD ± 15.41 (95% CI: 47.22, 69.28), translating to a ‘D’ grade. Educators demonstrated positive perspectives of new technology but found that AR presented more challenges than benefits. Recommendations focused on overcoming hardware difficulties and ensuring in‐depth educational content with reference to the cadaveric study. Mobile AR does not currently hold substantial value for anatomy education; however, the benefits of AR may be optimized using a head‐mounted display. Future research must consult all potential stakeholders to critically define how AR will provide measurable value for anatomy education.

## INTRODUCTION

Human anatomy is fundamental to the study of Medicine and Allied Health degrees.^1^, ^2^, ^3^, ^4^ Anatomy education is traditionally delivered through instructional lectures supported by textbooks, three‐dimensional (3D) models, and cadaveric specimens.^1^, ^2^, ^5^ Modern learning also incorporates educational technology (EdTech) involving online libraries, 3D‐printing, medical imaging, and interactive displays.^2^, ^4^, ^5^ Scientific advancements have increased the breadth of knowledge necessary for safe medical practice, and health science curricula have endeavored to incorporate this expanse through the integration of the anatomical and clinical sciences.^1^, ^2^, ^6^ This adds to the challenge of delivering an effective undergraduate anatomy education impacted by significant declines in teaching hours, specialty staff, and cadaveric donations.^1^, ^2^, ^7^, ^8^ The COVID‐19 pandemic furthered the need for flexible and accessible teaching methods which effectively engage students who are required to be exceptionally self‐motivated.^4^, ^9^

**FIGURE 1:**
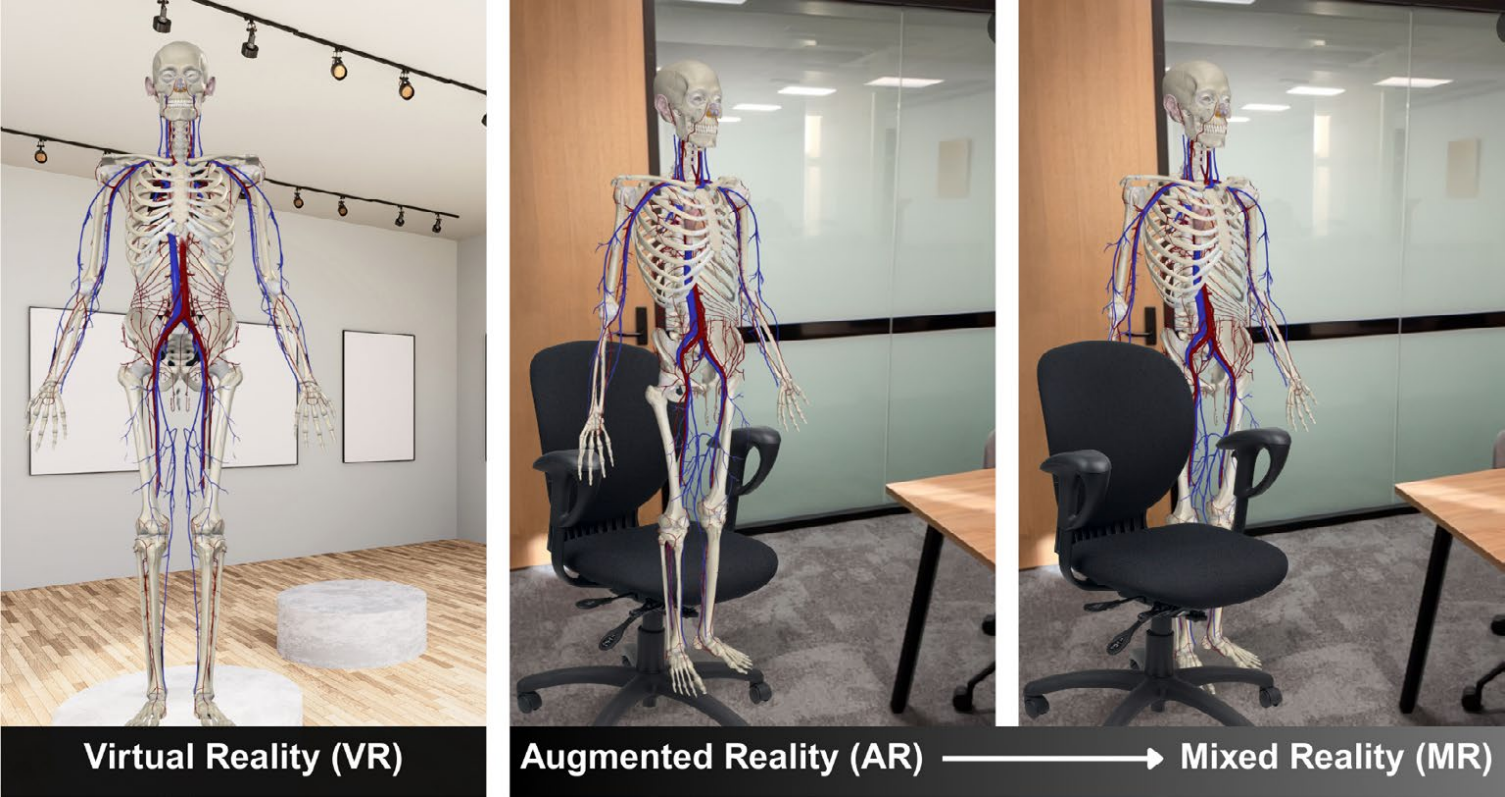
Virtual reality (VR) verses augmented reality (AR) and mixed reality (MR). Images courtesy of Complete Anatomy have been adapted. The first image is an illustrated example of VR where the digital anatomical model would be viewed in a fully digital environment via HMD. The second image depicts a basic form of AR where the digital model is overlaid on the real‐world environment. The third image illustrates MR as a higher level of AR, demonstrating real‐world integration of the digital model as it obeys the boundaries of the physical chair.

Augmented Reality (AR) has recently been explored to create simulated medical training where it is difficult or unethical to obtain the skills through real‐life scenarios, such as basic life support,^10^, ^11^ pediatrics,^12^, ^13^ remote practice,^14^, ^15^, ^16^ and surgical procedures.^17^, ^18^ AR merges digital objects with the real‐world using mobile devices such as tablets and smartphones, or a head‐mounted device (HMD) such as HoloLens.^19^ Other HMDs are designed to enable fully immersive virtual reality (VR) experiences. Modern terminology describes an AR continuum from basic digital overlays to more advanced mixed reality (MR) technologies that use HMDs or smart glasses to seamlessly integrate digital objects which act as an authentic part of the real environment.^20^ Figure 1 is designed to illustrate the distinctions between these digital realities.

### AR in medicine

AR in healthcare has benefited from advances in medical imaging, with technological development naturally aligned to applications in radiology and the operating theater.^21^ AR supports situational awareness in surgery by projecting images or data – usually presented on a stationary screen – through a HMD, so that the surgeon can remain task‐focused; optimizing surgical time, patient safety, and clinical costs.^22^, ^23^ AR has also demonstrated potential to enhance patient education and surgical planning^21^, ^24^; reduce fluoroscopic radiation dose through novel intraoperative guidance techniques^25^, ^26^, ^27^; and improve positioning accuracy in radiotherapy.^28^

### AR in anatomy education

AR EdTech has been developed to increase student engagement in complex subjects including anatomy education.^29^, ^30^, ^31^, ^32^ In a study by Weeks et al.,^32^ students using AR via HMDs to project a 3D computed tomography (CT) model alongside a real cadaver demonstrated higher learning outcomes than students who viewed the CT imaging via a laptop. Bork et al.^30^ found that students using AR were more inclined to participate in large‐group learning and may therefore benefit from the higher‐order thinking and communication skills associated with collaborative learning. Later research suggests that AR learning methods could be more time effective than anatomy labs.^31^ These studies demonstrate the potential of AR to alleviate the curricular challenges of anatomy education while introducing students to clinically relevant, modern technology.

### Theoretical rationale & research priority

Post‐pandemic, accessible learning methods are critical in supporting a diverse range of students across the undergraduate health sciences.^9^, ^33^, ^34^ The development of a national anatomy curricula is recommended to provide evidence‐based guidelines for consistent learning outcomes, and ensure students graduate with relevant anatomical knowledge which effectively transfers to safe and informed professional practice.^35^, ^36^ The increase in AR technology registered with the FDA^37^ demonstrates the rise of AR in Western healthcare; however, this is not yet reflected in Australian universities and healthcare settings.^2^, ^35^, ^38^ This is likely due to Australia's demand for high‐quality EdTech which supports best pedagogical practices.^29^, ^33^

AR learning tools support constructivist‐based learning as a multimodal tool which provides visual, interactive models of human anatomy overlaid or integrated with the real environment. These 3D digital models can be used as a tool for didactic demonstrations, group learning and individual study. Recent systematic reviews of AR for anatomy education report the positive impacts of AR EdTech.^38^, ^39^, ^40^, ^41^ The studies in these reviews present positive student feedback and show that AR learning methods enable equal or improved learning outcomes compared to traditional learning methods. It is difficult to draw definitive conclusions; however, due to highly heterogenous literature and the diversity of AR technology and terminology.^40^, ^41^, ^42^, ^43^, ^44^, ^45^ Few studies comment on the disadvantages of AR EdTech which include technological difficulties and adverse health effects such as eye strain and dizziness.^38^ At an institutional level, the necessary upskilling of educators to facilitate AR teaching methods, and the initial time and resource costs, present a more complex barrier to AR implementation.^29^ Studies have almost exclusively focused on the student opinion of AR without seeking the perspectives of key stakeholders such as industry professionals and university educators.^40^, ^41^, ^44^, ^46^ This has resulted in considerable biases and limited the strength of evidence necessary to reliably determine the effect of AR on learning outcomes.^40^, ^41^, ^42^, ^44^ It is immediately apparent that the perspectives of educators must be explored to strengthen the design of future studies, guide the development of AR EdTech, and examine the value of AR in a standardized anatomy curriculum.

### Research aims & objectives

The purpose of this exploratory study is to address the current research gap by exploring the perspectives of educators on using AR for anatomy education. By analyzing this valuable insight from the ’other half’ of the classroom, the researchers aim to explore the current value of AR in an anatomy curriculum according to Australian educators and provide recommendations for future research and development.

The main objective of this study is to reflect a cross‐section of Australian educators' perceptions of the benefits and challenges of using AR to deliver anatomy education in the undergraduate health sciences. The second objective is to quantify the usability of a professional AR application designed for learning human anatomy to provide comparison for future research and guide technological development.

## MATERIALS & METHODS

### Study design

This qualitative exploratory study followed a convergent mixed‐methods study design, commonly employed in health and educational research,^47^, ^48^ allowing a contextual in‐depth analysis of Australian university educators' current attitudes towards using AR to deliver anatomy education. Individual research sessions allowed educators to interact with digital anatomy via mobile AR before completing an online survey. The study was designed in consultation with an expert panel of six senior researchers and educators from the Faculty of Health Sciences (FHS) at Curtin University, with ethical approval from the Human Research Ethics Committee (HRE2023‐0261). The research was undertaken by a student‐researcher and two senior supervisors as part of an Honors year project.

The study is underpinned by an interpretive research approach, guided by constructivist grounded theory; acknowledging the student‐researcher as an active participant, and whose position as a non‐educator enables a unique and considered analysis of subjective data in the exploratory research.^49^ A constructivist paradigm supports this small study as an important contribution to the broader literature and provides a methodological guide to address bias through ethical analyses and respectful data interpretation.^49^ As a non‐educator, fulfilling the role of both primary researcher and analyst required ongoing reflexive practice. This was demonstrated through reflective note taking and ongoing peer review to limit bias and ensure research accountability. The study plan recognized that building trust with educators was critical to motivate open and honest participation. The research process prioritized authentic communication and respect for participant educators' wellbeing to ensure accurate data collection. It has been established that educators' opinion of AR technology for anatomy education has not been sufficiently explored. The literature review preceding this study therefore looked at AR compared to other methods for teaching anatomy.^41^ This mitigated inductive bias and supported the aim of exploring the current value of AR in anatomy education.

### Setting & participant recruitment

The target population was university educators in Western Australia (WA) whose teachings strongly incorporate human anatomy. Inclusion criteria required employment through Curtin University to satisfy ethical requirements and time constraints; therefore, research sessions were delivered at Curtin University's Perth campus. Exclusion criteria disallowed participation from any person who provided research guidance, including the advisory panel and specialist research consultants. Recruitment and data collection processes were concurrent from mid‐May to early July 2024. Anatomy educators were conveniently recruited from the FHS via email invitations using a discriminative snowball sampling method beginning with course co‐ordinators for maximum reach. This supported the credibility of invitations and facilitated respectful introductions.

### Sampling method

Guided by recommendations from Vasileiou et al.,^50^ researchers determined an acceptable sample size, concluding that 10 educators would provide adequate data for this study. The perspectives of 10 anatomy educators have the potential to provide considerable insight into the views of undergraduate anatomy educators in WA. Curtin University is 1 of only 9 universities in Australia to offer direct entry undergraduate medical programs and is the only university in WA facilitating on‐campus undergraduate degrees in Medicine, Pharmacy, Speech Pathology, Occupational Therapy, Medical Imaging, Radiation Therapy, and Paramedicine.^51^ The sample size restricts statistical analysis; however, the research aims to provide valid quantitative data for cross‐sectional or meta‐analysis. Limiting the sample supports the explorative nature of the research which foregoes pre‐determined hypotheses in favor of providing a detailed and in‐depth data analysis. Pragmatically, this ensured effective time management, prioritizing the quality and integrity of research primarily performed by an emerging researcher.

### Data collection tools

#### Mobile AR tool

Mobile AR was selected over HMDs to demonstrate AR in a familiar format, using the inbuilt cameras of a tablet device to overlay digital objects with the real‐world. The familiarity of mobile devices was intended to encourage engagement with the AR application rather than the hardware. Cost has been identified as a barrier to AR EdTech,^29^ and Apple's iPad,^52^ *10* ^*th*^ *Gen 10.9″*, purchased for $649AUD, minimized costs when compared with modern HMDs (HoloLens, $5599AUD^19^). Complete Anatomy (CA) *version 10.4* by 3D4Medical^53^ from Elsevier was chosen as an example of a professional and commercially available AR application designed for learning human anatomy. This combination of hardware and software provided accurate representation of modern AR EdTech, ensuring relevancy and validity in the research.

#### Descriptive survey tool

Qualtrics online software was used to build a cross‐sectional survey designed as an *online questionnaire*, and securely manage data analysis. Survey Part 1 gained informed consent and gathered contextual information including educators' previous experience with AR. Part 2.1 used the System Usability Scale (SUS)^54^ to provide a quantitative appraisal of user satisfaction. The SUS is a validated and reliable tool frequently used for evaluating the usability of AR EdTech, and suitable for comparing each stage of technical development.^45^, ^55^ The SUS comprises a list of ten statements with a 5‐point Likert scale response ranging from ‘strongly disagree’ to ‘strongly agree’. These responses are calculated to give a final score from 0–100 with scores above 68 considered to be above average.^55^ Part 2.2 presented four 5‐point Likert scale statements adapted from similar research by Kluge et al.^29^ which were designed to further categorize educators' general perceptions of AR. Part 2.3 used short‐answer feedback to determine the perceived benefits and challenges of AR for anatomy education.

### Data collection procedure

To encourage participation and engagement with the research, 30‐min individual research sessions were held at a time best suited to each educator. A table and ~1 m^2^ floorspace facilitated AR demonstrations, and a laptop was provided for participants to complete the survey in‐session.

#### Survey Part 1

Each research session began with the researcher (AW) providing a verbal overview of the research topic, the concept of AR, and the educator's role as a participant. A digital copy of the participant information sheet was included in the recruitment email which outlined the focus of the study, the expected time required, and how the participants' role in the research contributes to relevant outcomes. This was again provided through Part 1 of the survey which gained informed consent. Qualtrics generated a random number for each session which was used to link the researcher's reflective notes to the generated data. This maintained data anonymity while monitoring the research procedure for any issues which may impact response. It was explained as part of the consent process that these notes were impersonal and descriptive, and designed to limit bias by recording critical observations on any part of the procedure that may have influenced outcomes and provide proof of procedural congruence.

#### AR demonstration

The researcher initially ‘placed’ each AR model in the room for time efficiency, as preliminary tests found this feature frequently unreliable for first‐time users. While this likely increased usability scores compared to if the participant had placed the model, this decision supported the best use of time and was designed so that users could reflect on AR as a teaching tool without focusing on this specific problem with the application. SUS scores therefore reflect the subsequent interactions where educators used the mobile AR independently: rotating and scaling the anatomical model via the touchscreen, and physically moving the iPad to navigate through the model.

**FIGURE 2:**
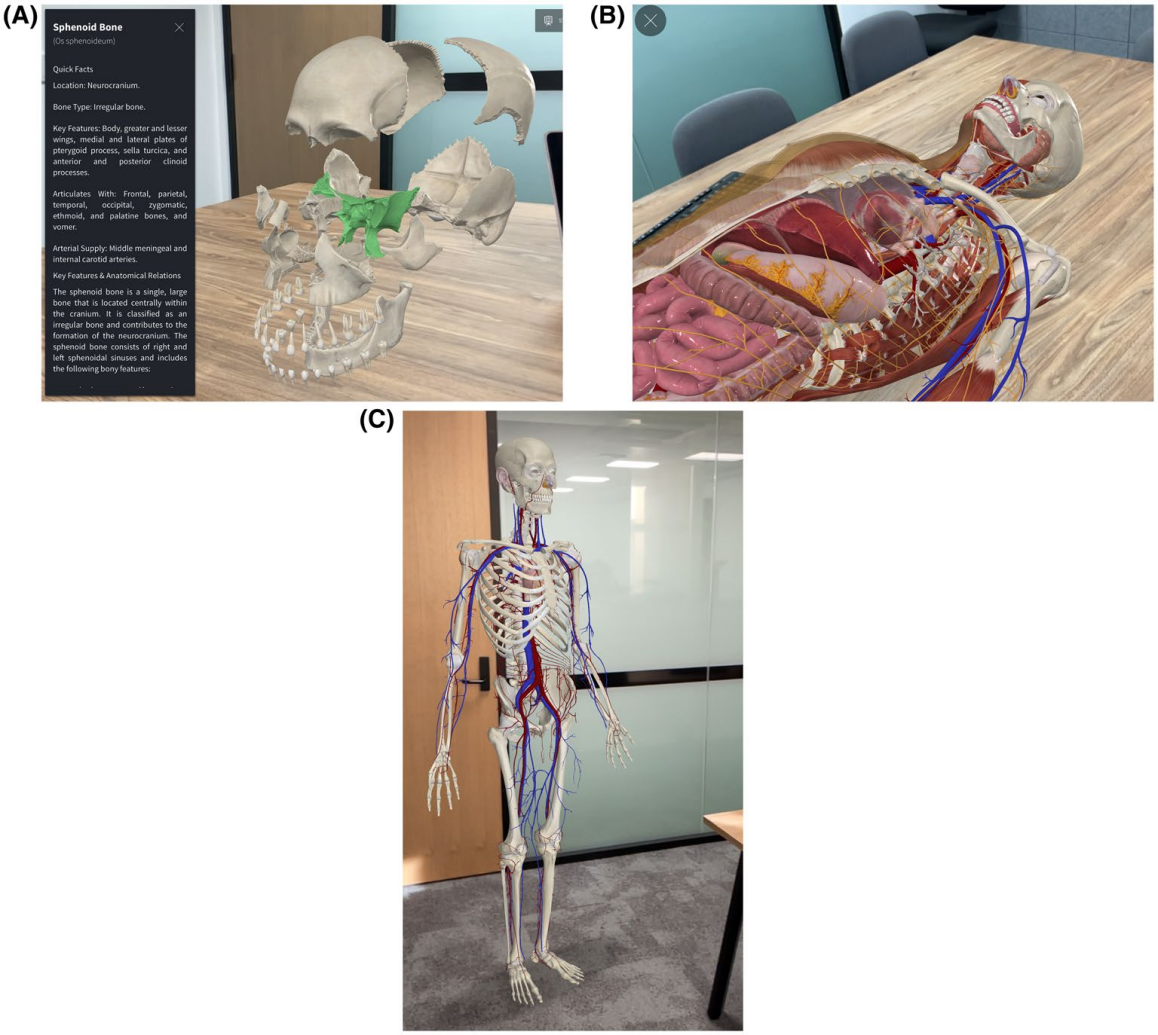
Anatomical models demonstrated via mobile AR. Images courtesy of Complete Anatomy demonstrate the digital anatomical models via mobile AR; (A) skull with explode feature and infographic, (B) full body dissection with beating heart, and (C) full skeleton with primary vasculature and beating heart.

Three AR models (Figure 2) were presented sequentially over approximately 15‐min; a human skull, a partially dissected body, and a skeleton featuring primary vasculature. Key features of CA's AR were introduced including, ‘explode’ which separates anatomical components; a ‘beating heart’ demonstrating cardiac function; and informatic displays. Participants were encouraged to ask questions or express concerns at any time.

#### Survey Part 2

Survey Part 2 was completed in the final 10‐min of the research session. Participants were not financially compensated; instead, motivation was offered through the opportunity to contribute to the broader discussion of pedagogical methods for anatomy education.

### Data analysis

Supported by constructivist grounded theory, iterative thematic analysis techniques were used to process the data.^49^ The primary researcher met with senior supervisors for frequent reflective discussion and at each point of data analysis to review code development. Formal presentation and critical feedback sessions were held with the expert panel to ensure research integrity.

Likert scale survey data was tabulated and graphed for comparative analyses, and SUS scores were calculated through Excel. Statistical analysis was not possible due to the small sample and exploratory intent of the study, however, ordinal data is provided for future research. Qualitative analysis of Educators' responses to short‐answer questions was conducted manually, line by line, to highlight emerging similarities. Guided by the research objectives, this inductive open coding technique grouped similar responses across all three short‐answer questions to develop categorized summations of educators' perceptions of AR including ‘*positive perceptions of AR*’, ‘*challenging aspects of AR*’, and ‘*recommendations for AR improvement*’. Educators' true wording was prioritized when defining categories, and outlying perspectives are noted in the results to minimize bias and demonstrate a comprehensive analysis. Axial coding was performed using visual diagrams, graphs and mind‐mapping to examine integrated themes emerging from this data and compare findings with the Likert scale data and SUS results for contextual data analysis. Iterative refinement of the full data set defined the final themes for discussion. Further comparison with the wider literature aimed to identify relationships which may be valuable for guiding future research into AR for anatomy education.

## RESULTS

### Participants

From 7 initial invitations, our sampling method provided 16 unique referrals over 2 months. Researchers contacted a total of 23 educators as potential participants. This resulted in the desired sample size of 10 educators; 7 respondents politely declined with reference to scheduling, and 6 invitations with follow ups received no response.

### Contextual data (Survey Part 1)

Nine educators were from the Curtin Medical School and 1 educator was from the School of Allied Health. Specialties included Medicine, Human Biology, Medical Radiation Science, Clinical Anatomy, Speech Pathology, Physiology and Biomedical Science. Teachings were focused on anatomy, clinical skills, and physiology. While limited to one response, educators were clearly specialized in several fields and used the ‘other’ option to list them. Experience with AR ranged from less than 24 h (*n* = 2), 4 weeks (*n* = 1), or 12 months (*n* = 2), and more than 1 year (*n* = 4). Most educators had prior experience using mobile AR and the Anatomage table (*n* = 7), followed by VR via HMD (*n* = 6), AR via HMD (*n* = 3), and AR smart glasses (*n* = 1). One educator did not record extent of experience but indicated prior AR use. While this discrepancy prohibited statistical data correlations, this educator's responses to survey Part 2.2 aligned their data to the ‘less than a year's experience’ group. Their data was assigned to this group indicated by “≈”.

### System Usability Scale (Survey Part 2.1)

**FIGURE 3:**
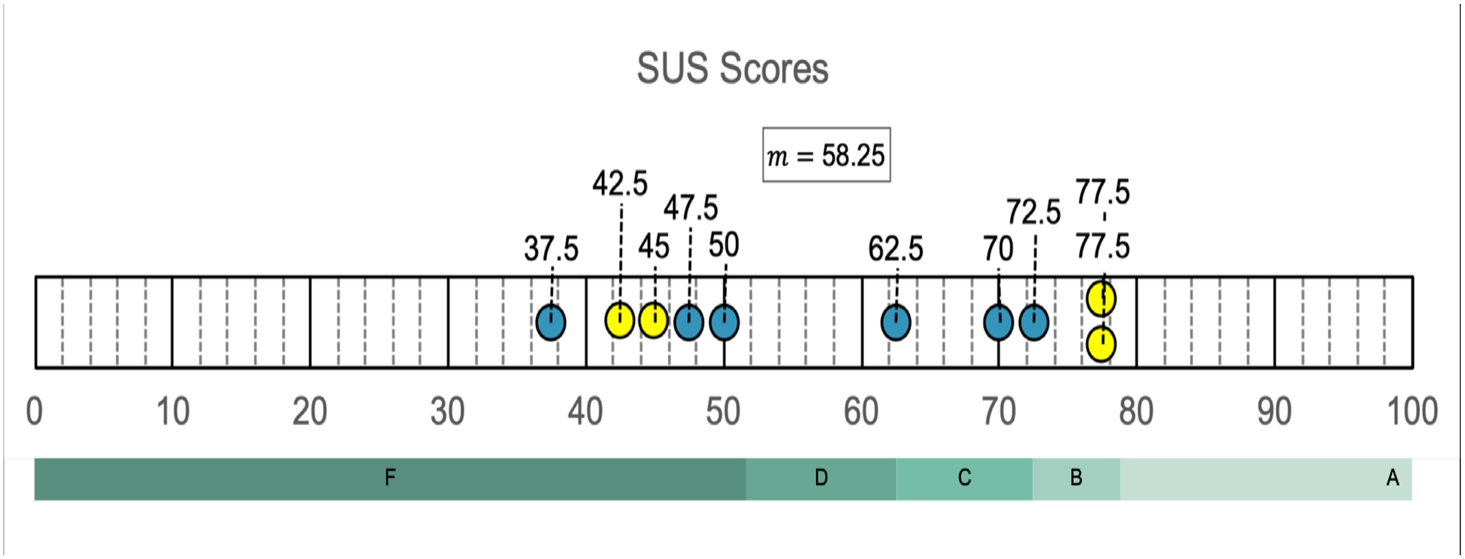
SUS results demonstrating AR usability (Survey Part 2.1). Scores from the SUS reflect the usability of Complete Anatomy's mobile AR feature using an iPad. SUS wording and calculations were determined according to the modern standard.^55^ Yellow icons represent the scores from educators with the most AR experience (more than one year). This is followed by the curved grading scale which provides a letter grade aligned to the SUS score.

The SUS survey returned a mean score of 58.25, SD ± 15.41 and a similar median score of 56.25 for the mobile AR feature using the CA application and iPad. A mean score of 58.25 is considered below the SUS average of ‘68’ and translates to a ‘**D**’ on the curved grading scale demonstrated in Figure 3. In this case, the mean and median scores are misleading as visual assessment showed a bimodal distribution of data. There were no commonalities found in the groups above and below these measures. User experience has previously been shown to correlate with usability scores,^55^ however, the individual SUS scores in this study did not correlate with the educators' experience in AR technology. The usability scores from the four educators who had more than a year's experience with AR were divided; two scores aligned to a grade **F**, well below average, and the other two scores gave an above average **B** grade (Figure 3). While this data cannot be generalized, it provides points for reflection or inclusion in future research.

### Quantified perceptions of AR (Survey Part 2.2)

**FIGURE 4:**
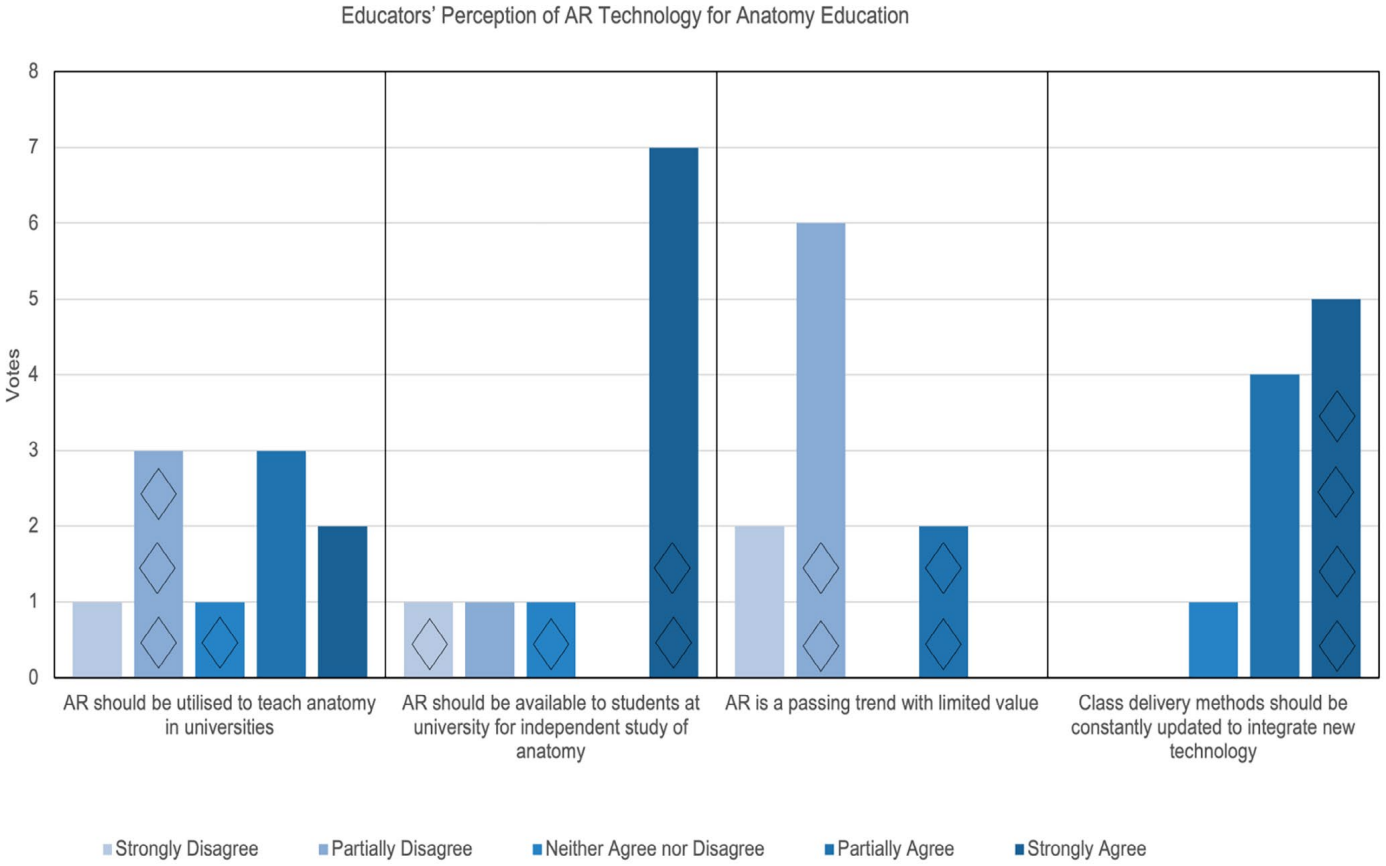
Educators' perception of AR for anatomy education (Survey Part 2.2). The above graph demonstrates educators' response to statements from strongly disagree to strongly agree. The diamond icons represent the responses from participants with the most AR experience (more than one year).

Quantitative analysis of educators' attitudes towards AR for anatomy education – presented in Figure 4 – demonstrate interesting differences when comparing educators with AR experience exceeding one year (Y+ group, *n* = 4) to those with less than a year's experience (<Y group, *n* ≈ 6). There is a clear difference of opinion between the groups on whether *AR should be utilized to teach anatomy in universities*. There is partial disagreement indicated by the Y+ group, while the majority of the <Y group indicated partial/strong agreement. Most participants strongly agreed that *AR should be available to students for the independent study of anatomy*, and strongly/partially disagreed that *AR is a passing trend with limited value*. The Y+ group responded with divided opinions for both statements (Figure 4). Despite more negative perceptions towards AR technology, the Y+ group all strongly agreed that *class delivery methods should be constantly updated to integrate new technology*, indicating positive attitudes towards technological advancement. The <Y group partially agreed with this statement.

### Perceptions of AR for anatomy education (Survey Part 2.3)

#### Positive perceptions of AR

Positive feedback was received from nine educators and one educator concluded that AR currently does not present any perceived benefits for learning anatomy where other digital methods cannot suffice. Educators appreciated AR as an accessible learning tool for independent study, which could offer an alternative learning method for students who cannot attend class.

To give students who cannot participate in class for one reason or another an opportunity to experience anatomy from a different perspective.

Educators praised CA's digital interactive features and contextual infographics for their potential to engage visual learners and customize anatomical models. The potential to visualize life‐size models and provide 3D spatial recognition, features unique to AR, received minimal mention.

#### Challenging aspects of AR

The challenging aspects of AR were centered on the limited usability of hardware and software. Educators often felt like they might drop the iPad and found it difficult to achieve stable interactions with the models. These difficulties, coupled with potential economic costs, were seen as barriers to accessibility and equal learning opportunities.

Relies on the student having good mobility ‐ may be an issue for those with accessibility requirements.

Ongoing funding to make sure all students have access to the technology.

Educators stated that they would ideally like to customize and dissect anatomical models in AR but also expressed concerns that these features would be time consuming to learn and teach without an exceptionally seamless interface. Educators praised technological progressions in anatomical illustration with realistic bony detail, however, stylised anatomical models were considered inadequate in comparison to cadaveric specimens. Without haptic feedback or the ability to replicate real‐life interactions, educators did not perceive sufficient value in AR.

Non‐bone tissues are poorly depicted when compared to their surgical or cadaveric appearance.

Does not replace the haptics of interacting with specimens. (i.e., reflecting muscles, placing tension on tendons to observe which joints they cross)

Cadaveric study was frequently referenced as a comparative standard. Caution seemed to be held in technological replication of anatomy, with respect to the value of anatomy labs.

Lack of actual, authentic wet specimens may limit student experience.

One of the biggest challenges would be incentivising students to continue to attend the wet labs. (because wet specimens are still the gold standard)

#### Recommendations for AR improvement

Recommended improvements to mobile AR focused on a more stable iPad experience with touch‐selective features including dissection, 360° rotations, labelling, and guidance points. One educator suggested incorporation of cross‐sectional anatomy with further potential to simulate medical imaging. Educators' responses emphasized the value of hands‐free and collaborative experience, and two responses suggested using VR. The data does not completely confirm if these educators' imagined VR in the true sense – a fully immersive digital environment via HMD – or if they imagined AR via HMD or other wearable device. However, it is most likely that this as a reference to HMDs as one response wished for a more tactile and ‘real’ experience and the other wanted to optimize group learning. These scenarios indicate a hands‐free AR experience which would increase the perception of reality and enhance group interactions. Educators also imagined the potential of hyper‐realistic, interactive models that could demonstrate detailed functionality.

…to be able to start superficially and work your way in to deeper layers to teach students perspective and anatomical relations.

Ability to see sections in functioning detail such as vocal folds still but also vibrating; also getting to detail of nerve bundles running in pyramidal tract for example.

Time efficiency was a concern for many educators. Feedback demonstrated frustration with technological design and development, highlighting the challenges of running an anatomy curriculum and the need for technology to support curriculum delivery.

…developers should look to take away some of the tedious aspects of education…All too often new technologies such as AR seem to concentrate on the “fun” and “interesting” activities of the teaching process. (which the educators in most cases, don't mind doing), rather than the day‐to‐day aspects of teaching

While educators indicated positive elements of AR and direction for future improvements, the challenges to AR for teaching anatomy suggest the need for specialized and thoughtful design to direct technological evolution. Final iterative analysis with full data integration presented three clear themes to direct future research and development: ‘*Optimal AR hardware’*; ‘*Cadaveric comparisons’*; and ‘*Value‐based design*’.

## DISCUSSION

This study explores the perspectives of ten Australian anatomy educators on the use of AR as a tool to deliver undergraduate anatomy education. Detailed feedback highlights educators' pedagogical values and establishes key considerations for future research and technological design.

### Optimal AR hardware

Discussion of AR in education must consider the critical impact of hardware.^39^, ^44^ There is a need to delineate between AR hardware delivery methods (e.g. mobile devices vs. HMDs) and recognize that the choice of hardware directly influences the desired benefits of AR. The use of mobile AR as an anatomy education research tool has been justified by its comparatively low cost, portability, and native familiarity to users, all of which establish potential for broad accessibility and scalability.^39^, ^44^ For similar reasons, our study utilized mobile AR via an iPad; however, our participating educators reported considerable difficulties holding the iPad and achieving stability when exploring the AR anatomical models.

Our research outcomes highlight how the practical challenges of mobile AR hardware may directly counteract some of AR's potential benefits. Physically holding the iPad means that users are not hands‐free to write notes or interact with real or digital models for simulated training. An Australian study by Moro et al.^56^ compared mobile AR to AR via HMDs for learning brain anatomy and found no difference in students' perceptions of usability after 6‐min lessons. Educators in our study physically explored larger AR models, holding the iPad for much longer, which may explain greater user difficulties. Moro et al.^56^ reflected that AR via HMD presented truer 3D representations which may be best for learning anatomy and physiology. HMD's user difficulties are well documented with multiple reports of cybersickness.^30^, ^56^ Other users have found difficulty holding their head still to properly view digital models, trouble communicating individual viewpoints, or expressed concern that the novelty experience of AR via HMD and the associated learning curve could cause excessive distraction.^30^, ^32^, ^56^ The quality of the software and interface design are also important variables to consider when justifying hardware selection. Commercial software can provide greater generalisability while in‐house software may be designed to cater to specialist learning requirements.

In the context of anatomy education, researchers must define which benefits of AR learning they wish to capitalize upon, and consider which hardware – mobile or HMD – will effectively facilitate these benefits.^57^ For example, Garzon et al.^58^ analyzed AR EdTech according to pedagogical grounding and found that the collaborative benefits of AR provide the strongest positive impact on learning outcomes. Researchers who require multi‐user engagement could consider that, while mobile AR offers greater portability at lower cost, AR via HMD allows hands‐free interactions with the real world, and increases the users' field of view for a more immersive or collaborative experience.^59^ This is already reflected in the dominant use of HMDs in healthcare for simulated training, surgical guidance, and remote learning scenarios.^59^ As evident in our educators' feedback, mobile AR may be better suited to support individual learning through portable 3D models or contextual information,^44^, ^60^ while HMDs, through hands‐free interaction, could optimize the collaborative and immersive benefits of AR for anatomy education.^59^, ^61^, ^62^

### Cadaveric comparisons

A major theme in our study is the comparison of AR to cadaveric learning methods. Educators expressed concerns that the pseudorealistic anatomical models presented via CA's AR feature were comparatively inadequate to cadaveric material. While cadaveric material is referenced by educators in this study and other literature as the gold standard for anatomy education, cadaveric learning is impacted by key challenges, including declines in body donation, specialist educators, and teaching hours, coupled with growing concerns around ethics and equal access.^2^, ^7^, ^63^

The concept of a single gold standard could be considered contradictive to modern teaching pedagogies which underscore multifaceted approaches to anatomy education. While educators rightly value authentic representation of human anatomy, resource allocation in anatomy curriculums will need to explore evidence‐based alternatives where access to cadaveric material is limited by rising costs and donor shortages.^4^, ^40^ The results of this study highlight that AR cannot replace cadaveric study nor sufficiently replicate ‘touch and feel’ interactions; however, AR has the potential to supplement limited anatomy lab access by simulating the visuospatial aspects of cadaveric dissection with multi‐user collaborative functions.^31^, ^64^

Commercial software is already making technological advancements to simulate dissection in AR, albeit with heavily stylised anatomical models and at the cost of significant financial investment.^65^, ^66^ Educators in our study noted cost as a major limitation of AR for anatomy education. Cost concerns, however, are directly linked to perceived value.^67^, ^68^ As demonstrated by our study, educators value anatomical detail, accuracy, and seamless interfacing. AR developers should consider leveraging advanced technology, such as photogrammetric visualization, to create photorealistic, purpose‐designed resources with genuine value whose benefits justify the necessary financial investment.^69^

Research outside of AR should examine the benefits of cadaveric study in a modern curriculum and define which health science disciplines or groups of students receive optimal benefit from this teaching method.

### Value‐based design

Educators in our study demonstrated divided opinions as to whether AR should be used to teach anatomy; however, most agreed that class delivery methods should integrate new technology. This strongly aligns with other Australian literature. For example, Kluge et al.^29^ found that educators at the University of Newcastle valued new technology and held similar concerns to our educators, including funding, content refinement, and potential impacts on current teaching and learning methods. Much like our study, Lee et al.^70^ found that anatomy educators did not perceive a sufficient level of detail and accuracy in CA's anatomical depictions and held diverse opinions of CA's usability. Lee et al.^70^ also surveyed students who, by contrast, highly rated CA's anatomical detail and usability. This is consistent with prior research which demonstrates that students hold predominantly positive perspectives of AR.^30^, ^56^, ^71^ In this context, our educators' concerns surrounding AR reiterate the divide in technological values between educators and students.

Diffusion of Innovation theory describes the people and characteristic variables that influence the gradual spread of an innovation ‐ in this case AR EdTech.^72^ The diffusion pattern is often represented by a bell curve which reflects the initial, small group of ‘innovators’, then ‘early adopters’, ‘early and late majority’ and ‘laggards’. The adoption of AR in higher education has rarely progressed beyond the ‘innovators’ and ‘early adopters’ who foresee a future in AR EdTech.^29^ Comparing their perspectives, it may appear that students and anatomy educators are at different stages; the students are ready to adopt current AR technology while educators appear more critical. Research indicates, however, that students are prone to erroneous perceptions of effective learning through ‘ease of use’.^57^, ^73^, ^74^ As reflected in the results of this study, experienced educators hold a more complex view of learning applications, valuing accurate and in‐depth educational content, seamless interfacing, and technological features which support curriculum delivery.^70^ Australian educators clearly value new technology; however, implementation of AR EdTech must not be at the expense of proven teaching methods or further burden faculty time and resources.^29^

Current AR innovations have largely focused on the student experience and neglected to design sustainable infrastructure to support adoption at an institutional level.^41^, ^46^ Rather than students' or educators' attitudes reflecting different stages of a seemingly inevitable diffusion of AR EdTech, the research highlights a difference in values and pedagogical understanding, and opportunities for technological improvement. The potential benefits of AR cannot hold value without holistic technological design and implementation plans, both of which must consider teaching delivery, current curricular workloads, future costs, and adaptations for specific educational contexts.^29^, ^46^

Future technological development in anatomy education may be best directed through value‐based design, incorporating the more nuanced perspectives of expert anatomy educators. Value‐based design principles support decision making in healthcare, education, and technology.^75^, ^76^, ^77^ Their use would direct energy away from finding ‘what works’ and instead require the involvement of all affected stakeholders to establish sustainable and flexible technological solutions which move beyond novelty or immediate scalability.^78^, ^79^ Current research is heavily focused on medical students and future AR designs for anatomy education may benefit from exploring applications for specialist anatomical studies in the wider undergraduate health sciences.^40^, ^80^

## LIMITATIONS

This research holds several limitations. Our survey did not force answers, and this prevented full recording of educators' duration of previous AR experience. In future, a prompt would be useful to avoid blank answers and permit a full data set. All ordinal data recorded in our study provide descriptive benchmarks for future comparisons; however, our sample size restricted statistical calculations. The SUS data would be further strengthened with a more direct comparative analysis, and we hope this exploratory study provides a comparison for future research. While our sample size may be considered small, it was designed for detailed qualitative analysis and, with reference to the declining numbers of anatomists and specialist educators, it reflects the sampling results of similar studies.^29^, ^70^ Examining the results of a 2022 review of AR in medical education, including 54 studies over 20 years, found that only 2 studies collectively included 8 university educators as participants.^81^ Our own review of the research since did not find any studies that had recruited educators as participants.^41^ In this context, our study's sample size of 10 anatomy educators provides a valuable contribution to the research.

In future, more participants may be recruited through an interview‐based method of data collection which reduces read‐write burden.^82^ An interview‐based method would have also allowed clarification of participants' meaning of ‘VR’ which was assumed as a reference to HMDs. While this did not greatly impact our study, it highlights potential ambiguity when discussing digital reality technologies and supports the call for agreed terminology.^20^ A significant outcome of our study was the influence of hardware on the potential benefits of AR, and with hindsight, this could have been more considered in the study's design.

It has been suggested that a positive selection bias may be apparent when participants are interested in digital reality technology.^29^ This is potentially reflected in our results, which show that educators agreed that *class delivery methods should be constantly updated to integrate new technology*. Outcomes that demonstrate educators' respect for the more traditional method of cadaver study and their critique of AR technology suggest that this bias is not a cause for concern in our study.

Most participants in this study were from the Curtin Medical School and provided valuable insight; however, results may have demonstrated more diversity if our sample was expanded to include more perspectives from the School of Allied Health. Furthermore, transferability of this study is limited and cannot directly represent the opinions of all Australian anatomy educators. Anatomy educators at Australian universities where AR is integrated in the curriculum through pilot programs may have more diverse or definitive opinions. Internationally, where software education has been developed for the consumer market, educators may hold an overall positive view.

## CONCLUSION

The findings of this study demonstrate that the value of AR, at least in mobile form, does not currently demonstrate sufficient value as a pedagogical tool for anatomy education. Reference to cadaveric material is consistent in the educators' feedback and reflect the value of accurate and detailed representation of anatomy, while demonstrating that the ongoing discussion of cadaveric study is still highly relevant in Australia. However, these educators value new technology and recommend future AR applications prioritize accurate and specialized educational content with technological features designed to support curriculum delivery. A national anatomy curriculum should go beyond standardized competencies to include guidelines for technological development which detail the importance of engaging with all stakeholders beyond the ‘end user’. Collaboration between universities, industry, and government would ensure funding, and allow resource sharing or commercialisation with adherence to national privacy laws.^83^ Future research should consider the diversity of user groups in the undergraduate health sciences and the extended potential of designing AR for Allied Health disciplines. Going forward, researchers must critically define how AR technology will provide sufficient pedagogical value and actively address the current challenges of anatomy education.

## AUTHOR CONTRIBUTIONS

**Ally Williams:** Conceptualization; methodology; investigation; validation; formal analysis; writing – original draft; writing – review and editing. **Zhonghua Sun:** Conceptualization; methodology; supervision; validation; project administration; writing – review and editing; resources. **Mauro Vaccarezza:** Methodology; conceptualization; supervision; resources; writing – review and editing.

## FUNDING INFORMATION

This research received no external funding.

## CONFLICT OF INTEREST STATEMENT

The authors report that there are no competing interests to declare.

## ETHICS APPROVAL STATEMENT

Ethical approval for this study was obtained from the Human Research Ethics Committee (HRE2023‐0261).

## Supporting information


**Data S1:** Supporting Information.
