# Supplementary material for: Augmented reality for teaching undergraduate human anatomy: An educators' perspective
Source: Anat Sci Educ. 2026 Mar 8;19(5):671–83. doi: 10.1002/ase.70214 (PMC13184588; doi:10.1002/ase.70214)
Supplement: Supplementary file 1 — Data S1: Supporting Information. [file ASE-19-671-s001.docx]

**SUPPORTING INFORMATION -** **ONLINE QUESTIONNAIRE**

**Part 1.1 Background (5 Questions)**

Q1. Please indicate your School

- ____________________

Q2. Please indicate your educational specialty

- Medical Radiation Sciences
- Medicine
- Nursing
- Other: ____________________

Q3. Please indicate the focus of the classes you teach.

- Clinical skills
- Anatomy
- Pathology
- Medical informatics (Merging of healthcare and information technology)
- Other: ____________________

Q4. Please indicate your experience using VR or AR either personally or professionally.

- None
- Less than 24 Hours
- Less than 7 Days
- Less than 4 Weeks
- Less than 12 Months
- 1 Year or more

Q5. Please indicate which extended realities hardware you have experienced either personally or professionally.

- None
- VR Headset
- AR Headset
- AR Smart Glasses
- AR Mobile (eg. Smartphone, Tablet, iPad)
- AR Anatomage Table
- AR Magic Mirror
- Other: _________

**Part 2.1 System Usability Scale (10 Questions)**

*(Answers rated 1-5: strongly disagree, partially disagree, neither agree nor disagree, partially agree, strongly agree)*

Q1. I think that I would like to use this product frequently.

Q2. I found this product unnecessarily complex.

Q3. I thought this product was easy to use.

Q4. I think that I would need the support of a technical person to be able to use this product.

Q5. I found the various functions in this product were well integrated.

Q6. I thought there was too much inconsistency in this product.

Q7. I would imagine that most people would learn to use this product very quickly

Q8. I found this product very cumbersome (awkward) to use.

Q9. I felt very confident using this product.

Q10. I needed to learn a lot of things before I could get going with this product.

**Part 2.2 Perception of AR Technology in Anatomy Education (3 Questions)**

*(Answers rated 1-5: strongly disagree, partially disagree, neither agree nor disagree, partially agree, strongly agree)*

Q1. AR should be utilised to teach anatomy in universities

Q2. AR should be available to students at university for independent study of anatomy

Q3. AR is a passing trend with limited value

Q4. Class delivery methods should be constantly updated to integrate new technology

**Part 2.3 Short-Answer Qualitative (3 Questions)**

Q1. What do you perceive are the best aspects of AR for delivering anatomy education?

Q2. What do you perceive are the challenging aspects of the AR for delivering anatomy education?

Q3. What other features do you think could be added to enhance AR for anatomy education?
